# Prediction of Potential Associations Between miRNAs and Diseases Based on Matrix Decomposition

**DOI:** 10.3389/fgene.2020.598185

**Published:** 2020-11-16

**Authors:** Pengcheng Sun, Shuyan Yang, Ye Cao, Rongjie Cheng, Shiyu Han

**Affiliations:** Department of Obstetrics and Gynecology, The Fourth Affiliated Hospital of Harbin Medical University, Harbin, China

**Keywords:** miRNA, matrix decomposition (MFMDA), endometrial cancer, miRNA–disease association, computational prediction model

## Abstract

It is known that miRNA plays an increasingly important role in many physiological processes. Disease-related miRNAs could be potential biomarkers for clinical diagnosis, prognosis, and treatment. Therefore, accurately inferring potential miRNAs related to diseases has become a hot topic in the bioinformatics community recently. In this study, we proposed a mathematical model based on matrix decomposition, named MFMDA, to identify potential miRNA–disease associations by integrating known miRNA and disease-related data, similarities between miRNAs and between diseases. We also compared MFMDA with some of the latest algorithms in several established miRNA disease databases. MFMDA reached an AUC of 0.9061 in the fivefold cross-validation. The experimental results show that MFMDA effectively infers novel miRNA–disease associations. In addition, we conducted case studies by applying MFMDA to three types of high-risk human cancers. While most predicted miRNAs are confirmed by external databases of experimental literature, we also identified a few novel disease-related miRNAs for further experimental validation.

## Introduction

Non-coding RNA (ncRNA) is a type of RNA that cannot be translated into protein. Although ncRNA cannot be translated into protein, its target gene can be regulated at the post-transcriptional level, thereby affecting disease (Hammond, 2015). A large amount of research evidence indicates that mutations and disorders of ncRNA are important causes of disease. Therefore, the identification of disease-related ncRNA has become an important topic in the field of biological research in recent years. ncRNA is a huge family and can be divided into housekeeper ncRNA and regulatory ncRNA (Kapranov et al., 2007; Lindsay et al., 2017). Housekeeping ncRNA is closely related to cell function, mainly involved in gene translation, gene splicing, gene modification, etc. The main function of regulating ncRNA is to regulate the expression level of genes. As regulatory ncRNA, miRNA is a class of non-coding single-stranded RNA molecules with a length of 22 nucleotides encoded by endogenous genes. They participate in the regulation of post-transcriptional gene expression in animals and plants (Taft et al., 2007; Chen et al., 2015). So far, 28645 miRNA molecules have been found in animals, plants, and viruses. Most miRNA genes exist in the genome in the form of single copies, multiple copies, or gene clusters (Wang and Chang, 2011).

In recent years, more and more studies have shown that miRNA plays a huge role in the process of cell differentiation, biological development, and disease development, which has also attracted more researchers’ attention (Xu et al., 2004; Jiang et al., 2012; Li et al., 2014; Kang et al., 2020). With further in-depth research on the mechanism of action of miRNA, and the use of the latest high-throughput technologies such as miRNA chips to study the relationship between miRNA and disease, people will make higher eukaryote gene expression regulation Network understanding has improved to a new level (Cui et al., 2006). This will also make miRNA a new biological marker for disease diagnosis; it may also make this molecule a drug target, or simulate this molecule for new drug development, which will likely provide a new treatment for human diseases (Goh et al., 2016).

However, using biological experiments to identify disease-associated miRNAs is expensive and time-consuming, and it is blind. Therefore, there is an urgent need for simple and effective computational prediction models for predicting disease-related miRNAs. With the rapid development of high-throughput sequencing technology, more and more omics data are published, which also provides data support for the study of computational prediction models (Yi et al., 2017). In recent years, many scholars have proposed some effective computational models for predicting miRNA related to complex diseases. According to their respective implementation strategies, we can roughly divide these methods into machine-based computational prediction methods and network-based computational prediction methods (Zou et al., 2016).

Machine learning-based computational prediction methods predict the association of potential miRNAs with the disease can be divided into supervised-based machine learning methods and semi-supervised-based machine learning methods. The method based on supervision is mainly based on labeling sample set and label-less sample set to construct a machine learning model. Jiang et al. extracted feature sets based on known and unknown associations for training support vector machine (SVM) classifiers to predict potential miRNAs and disease associations, and achieved comparative prediction performance through cross-validation (Maly et al., 2019). Qu et al. (Zou et al., 2015) developed a new calculation method based on the KATZ model to predict MiRNA disease association (KATZMDA) by integrating multiple data sources. Based on the known miRNA–disease association in the HMDD database, Li et al. (2017) developed a MiRNA–disease association prediction model (MCMDA) called the matrix completion algorithm. The MCMDA model uses a matrix completion algorithm to update the adjacency matrix of known miRNA–disease associations and further predict potential associations. Xu et al. (Chen et al., 2018) proposed a method based on low-rank matrix completion to predict miRNA–disease association (LRMCMDA). LRMCMDA first constructs negative samples based on known associations, and then uses a low-rank matrix to complete the model to infer all miRNA and disease associations. Cross-validation shows that the model has obtained reliable prediction performance. However, although this supervised machine learning method uses different ways to define negative sample data, it is difficult to deal with the actual situation in any way, which will affect the prediction performance. In order to overcome this limitation, Chen and Yan (2014) proposed a least-squares-based semi-supervised machine learning method for predicting the association of potential miRNAs with disease, referred to as RLSMDA for short. The RLSMDA method constructs a continuous classifier function, and the predicted value reflects the probability score between specific miRNAs and specific diseases. This method can obtain the predicted values of all miRNAs and diseases at the same time, and does not require negative sample data. In addition, the RLSMDA method can also predict miRNAs associated with isolated diseases. Xu et al. (2019) designed a set of probabilistic matrix decomposition algorithms by integrating the similarity of miRNAs with diseases, using known correlation matrices and integrated similarity matrices to identify miRNAs that are potentially related to diseases. Luo et al. (2017) proposed a semi-supervised method called KRLSM to reveal the association between miRNA and disease. Machine learning has been a hot topic in recent years, and some machine learning methods can be used to solve this problem. Despite the outstanding contributions made by existing methods, there is still room for improvement in prediction accuracy.

In addition to machine learning-based methods, network-based methods to predict disease-related miRNAs have also attracted the attention of many researchers. Such methods are mainly based on a common biological hypothesis, “miRNAs with similar functions are more likely to be associated with disease phenotypes with similar functions, and vice versa” (Jiang et al., 2010). Based on this basic assumption, Jiang et al. proposed a new method that uses Bayesian models to integrate genomic data to rank disease-related miRNAs. Chen et al. (2012) adopted the global network similarity measure and proposed an improved restart-based random walk model (RWRMDA) to predict the association between miRNAs and disease. Yet, this method is not suitable for predicting new disease-related miRNAs. Xuan et al. (2013) integrated the information entropy of disease entries and the similarity of disease phenotypes to measure the functional similarity of diseases and miRNAs, and gave greater weight to miRNAs belonging to the same family or the same cluster class, and proposed a k-nearest neighbor prediction model (HDMP) is used to predict disease-related miRNAs. This method has obtained reliable prediction performance, but also cannot predict miRNAs associated with isolated diseases. Later, Xuan et al. (Banys-Paluchowski et al., 2015) further proposed the MIDP method based on random walk. In this model, by assigning different weights to known and unknown nodes, the prior information of the topology is effectively integrated. In addition, the extended conversion on the double-layer network of miRNA diseases makes it possible to predict miRNAs associated with isolated diseases. You et al. (2017) proposed a path-based miRNA–disease association (PBMDA) prediction model by integrating known human miRNA–disease associations, miRNA functional similarities, disease semantic similarities, and Gaussian interaction profiles for miRNA and disease similarities. The model constructs a heterogeneous graph composed of three interrelated subgraphs, and further uses a depth-first search algorithm to infer potential miRNA–disease associations. The results show that reliable performance is obtained. Gu et al. (2016) created a network consistency projection algorithm to identify potential associations (NCPMDA) by integrating similarity networks and association networks. The biggest advantage of these methods is that they can predict isolated miRNAs associated with disease, but the performance obtained is not very satisfactory.

Although research on miRNA disease association prediction models has made some progress, there is still room to further improve the prediction performance of the model. In this study, we propose a predictive model called matrix decomposition, which fully considers the similarity between miRNAs and the similarity between diseases. In order to evaluate the effectiveness of MFMDA, we tested it using a global fivefold and local LOOCV framework. MFMDA is superior to the benchmark algorithm used for comparison, and achieves reliable performance in the framework of fivefold CV and local LOOCV (AUC 0.9061 and 0.7933) in the HMDD (V2.0) data set. To further prove the superiority of MFMDA, we analyzed three common diseases. Based on the analysis of the test results, we can find that 18 of the top 30 potential miRNAs related to the three diseases predicted by MFMDA have been confirmed by other databases.

## Materials and Methods

### Human Disease–miRNA Interactome Network

In the past few decades, as the technology has matured, a large number of omics data have been published, including a large number of pairs related to miRNA diseases. Here, we use the known miRNAs and disease-associated data set HMDD V2.0 as the benchmark dataset (Huang et al., 2019a). The data set contains 495 miRNAs and 383 diseases and 5430 experimentally verified human-disease-related pairs. We use the adjacency matrix *A* to represent this confirmed association. Specifically, if the disease *d*(*i*) was previously associated with miRNA *m*(*j*), the value of *A*_*ij*_ is 1; otherwise, the corresponding position is set to 0.

### miRNA Functions Similarly

Based on previous research, it is not difficult to find that miRNAs with similar functions are more likely to be related to similar diseases (Wang et al., 2010). Under this assumption, the miRNA functional similarity score was calculated^1^. Therefore, we constructed a functional similarity matrix FS between miRNAs based on these data, where *FS*(*m*(*i*),*m*(*j*)) represents the similarity between miRNA *m*(*i*) and another miRNA*m*(*j*).

### Disease Semantic Similarity

Semantic similarity is a common way to express the similarity of diseases in this field. MFMDA uses a layered directed acyclic graph (DAG) to calculate the similarity between two diseases (Wang et al., 2010). Specifically, for disease d, let *DAG*_*d*_ = (*d*,*T*_*d*_,*E*_*d*_) be a DAG, where *T*_*d *_ represents the ancestor node set of *d* (including itself) and *E*_*d*_ represents the hierarchical connection between diseases defined by the MeSH disease tree structure of the National Library of Medicine. For any *t* ∈ *T*_*d*_, MFMDA defines the semantic contribution of disease *t* to *d* as:

(1) $$D_{d}\left(t\right)=\left\{ \begin{array}{c} 1\;ift=d\\ max\left\{\Delta \times D_{d}\left(t^{\prime }\right)|t^{\prime }\in childrenoft\right\}\;ift\neq d \end{array}\right.$$

Where Δ is the semantic decay factor, which is set to 0.5 in the iterative equation according to previous researches (Dong et al., 2019; Marcuello et al., 2019). Therefore, the semantic similarity between the diseases *d*_*1*_ and *d*_*2*_ can be defined as:

(2) $$D\left(d_{i},d_{j}\right)=\frac{\sum_{t\in T_{d_{i}}\cap T_{d_{j}}}\left(D_{d_{i}}\left(t\right)+D_{d_{j}}\left(t\right)\right)}{\sum_{t\in T_{d_{i}}}D_{d_{j}}\left(t\right)+\sum_{t\in T_{d_{j}}}D_{d_{j}}\left(t\right)}\;$$

### Gaussian Similarity of miRNA and Disease

Among various similarity measurement algorithms, Gaussian similarity is a very good measurement method, which has been widely used in various fields. Let *VP*(*m*_*i*_) be the vector related to miRNA *m*_*i*_in Y,  i.e., the *i*^*th*^ column of Y. Then, the Gaussian similarity between the diseases *m*_*i*_ and *m*_*j*_is calculated as follows:

(3) $$KM\left(r_{i},r_{j}\right)=exp\left(-\gamma_{m}{||VP\left(r_{i}\right)-VP\left(r_{j}\right)||}^{2}\right)$$

Where γ_*m*_ is the adjustment parameter of the bandwidth (van Laarhoven et al., 2011). The update rule of parameter γ_*m*_ is as follows:

(4) $$\gamma_{m}=\gamma_{m}^{{}^{\prime }}/\left(\frac{1}{nm}\sum_{i=1}^{nm}{||VP\left(r_{i}\right)||}^{2}\right)$$

Similarly, the Gaussian similarity between miRNAs can be defined as follows:

(5) $$KD\left(d_{i},d_{j}\right)=exp\left(-\gamma_{d}{||VP\left(d_{i}\right)-VP\left(d_{j}\right)||}^{2}\right)$$

(6) $$\gamma_{d}=\gamma_{d}^{\prime }/\left(\frac{1}{nd}\sum_{i=1}^{nd}{||VP\left(d_{i}\right)||}^{2}\right)$$

### Integrated Similarity for Diseases and miRNAs

In order to obtain a more comprehensive disease similarity, the semantic similarity of the disease is combined with the Gaussian interactive contour kernel similarity through the following piecewise function to obtain the final similarity between the diseases:

(7) $$S_{d}\left(d_{i},d_{j}\right)=\left\{ \begin{array}{c} \begin{array}{cc} D\left(d_{i},d_{j}\right) & d_{i}andd_{j}hassemanticsimilarity \end{array}\\ \begin{array}{cc} KD\left(d_{i},d_{j}\right)\; & otherwise \end{array} \end{array}\right.$$

Similarly, the similarity between miRNAs can also be redefined as:

(8) $$S_{m}\left({\text{m}}_{i},m_{j}\right)=\left\{ \begin{array}{c} \begin{array}{cc} FS\left({\text{m}}_{i},m_{j}\right) & r_{i}andr_{j}hasfunctionalsimilarity \end{array}\\ \begin{array}{cc} KM\left({\text{m}}_{i},m_{j}\right)\; & otherwise \end{array} \end{array}\right.$$

### MFMDA

Matrix factorization (MF) is an effective technique that has been widely used in data representation (Huang and Zheng, 2006; Hosoda et al., 2009; Zheng et al., 2009; Xu et al., 2020). It aims to find two matrices whose product provides the best approximation to the original matrix. Given a miRNAs–diseases association matrix, MF can be decomposed into two matrices *Y* = *R*^*n*×*m*^, that is, *W* ∈ *R*^*n*×*k*^ and *H* ∈ *R*^*m*×*k*^, and *Y*≈*UV^T^*. Here, we use mathematical formulas to express the potential association prediction problem between diseases and miRNAs as the following objective function:

(9) $$\min_{U,V}{||I\cdot \left(Y-WH^{T}\right)||}_{F}^{2}$$

where $||\cdots |{|}_{F}^{2}$ represents the Frobenius norm and ⋅ denotes the Hadamard product of two matrices, that is, the multiplication of the corresponding elements of the matrix, and *I*_*ij*_ = 0 if the entry (*i*,*j*) in *Y* is missing, and 1 otherwise.

The standard MF in Eq. 2 is just to find two matrices, and their product tries to approximate the original matrix. However, the effects caused by the similarity between miRNAs and diseases are ignored. Suppose the functions of the two miRNAs are very similar, and at the same time, the diseases implicitly learned that they should have a similar distance in the vector space. The diseases dimension is the same. For the same reason, the miRNAs size can also use this idea to constrain the drug’s implicit representation. That is, if the two diseases are similar, the distance of the miRNAs in the low-dimensional vector space should also be small.

(10) $$\begin{array}{c} \min_{U,V}{||I\cdot \left(Y-WH^{T}\right)||}_{F}^{2}+\lambda_{l}\left({||W||}_{F}^{2}+{||H||}_{F}^{2}\right)\\ +\lambda_{v}\sum_{i,p=1}^{n}{||w_{i}-w_{p}||}^{2}S_{i,p}^{m,*}\\ +\lambda_{d}\sum_{j,k=1}^{m}{||h_{j}-h_{k}||}^{2}S_{j,k}^{d,*} \end{array}$$

where λ_*l*_, λ_*d*_, and λ_*v*_ are the regularization coefficients; *w*_*i*_ and *h*_*j*_ are the *i*th and *j*th rows of W and H, respectively. *S*^v*^ is the hidden social similarity between miRNAs and *S*^d*^ is the hidden social similarity between diseases.

#### Optimization

In order to solve the local optimal solution problem of Eq. 3, we use the gradient descent algorithm to solve. According to the nature of the Frobenius norm, the corresponding Lagrange function *L*_*E*_ of Eq. 2 can be redefined as:

(11) $$\begin{array}{c} L_{E}=Tr\left(I\cdot \left(YY^{T}-2*YHW^{T}+WH^{T}HW^{T}\right)\right)+\\ \lambda_{l}Tr\left(WW^{T}\right)+\lambda_{l}Tr\left(HH^{T}\right)+\lambda_{m}Tr\left(W^{T}L_{m}W\right)+\\ \lambda_{d}Tr\left(H^{T}L_{d}H\right)+Tr\left(\emptyset W^{T}\right)+Tr\left(\psi H^{T}\right) \end{array}$$

where *Tr*(⋯) represents the trace of a matrix; *L*_*m*_ = *D*_*m*_−*S*^m*^ and *L*_*d*_ = *D*_*d*_−*S*^d*^ are the graph Laplacian matrices for *S*^m*^ and *S*^d*^, respectively; and *D*_*m*_ and *D*_*m*_ are the diagonal matrices whose entries are row (or column) sums of *S*^m*^ and *S*^d*^, respectively.

The partial derivatives of the above functions with respect to W and H are:

(12) $$\begin{array}{c} \frac{\partial L_{E}}{\partial W}=-2YH+2WH^{T}H+2\lambda_{l}W+2\lambda_{m}L_{m}W+\emptyset \\ \frac{\partial L_{E}}{\partial H}=-2Y^{T}W+2HW^{T}W+2\lambda_{l}H+2\lambda_{d}L_{d}H+\psi \end{array}$$

According to the solution conditions of Karush–Kuhn–Tucker (KKT) (Facchinei et al., 2013), we can make ∅_*ik*_*w*_*ik*_ = 0and ψ_*jk*_*h*_*jk*_ = 0, thus obtain the following equations for *w* and *h*:

(13) $$\begin{array}{c} -{\left(YH\right)}_{ik}w_{ik}+{\left(WH^{T}H\right)}_{ik}w_{ik}+{\left(\lambda_{l}W\right)}_{ik}w_{ik}+\\ {\left(\lambda_{m}\left(D_{m}-S^{m,*}\right)W\right)}_{ik}w_{ik}=0\\ -{\left(Y^{T}W\right)}_{jk}h_{jk}+{\left(HW^{T}W\right)}_{jk}h_{jk}+{\left(\lambda_{l}H\right)}_{jk}h_{jk}+\\ {\left(\lambda_{d}\left(D_{d}-S^{d,*}\right)H\right)}_{jk}h_{jk}=0. \end{array}$$

Therefore, we get the *w*_*ik*_ and *h*_*jk*_ update rules as follows:

(14) $$\begin{array}{c} w_{ik}=w_{ik}\frac{{\left(YH+\lambda_{m}S^{m,*}W\right)}_{ik}}{{\left(WH^{T}H+\lambda_{l}W+\lambda_{m}D_{m}W\right)}_{ik}}\\ h_{jk}=h_{jk}\frac{{\left(Y^{T}W+\lambda_{d}S^{d,*}H\right)}_{jk}}{{\left(HW^{T}W+\lambda_{l}H+\lambda_{d}D_{d}H\right)}_{jk}} \end{array}$$

**FIGURE 1:**
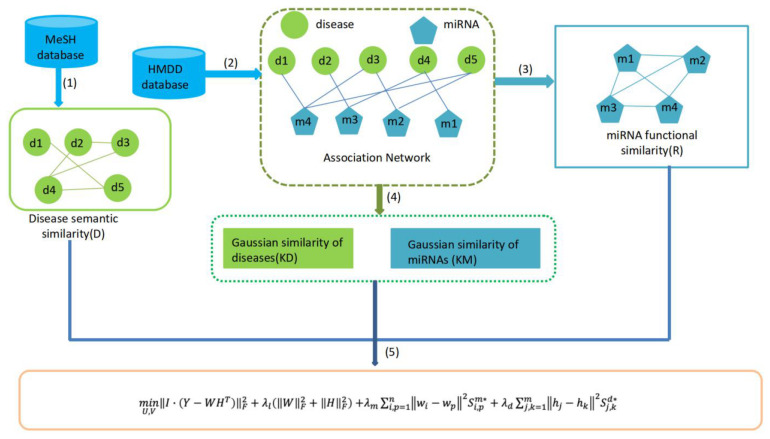
Diagram of MFMDA for predicting potential miRNA–disease associations.

The matrices W and H are updated based on Eq. 3 until convergence. Finally, we can obtain the predicted miRNAs–diseases association matrix as *Y*^∗^ = *WH^T^*, and determine the priority of potential miRNAs and disease according to the value in the matrix *Y*^∗^. In principle, the miRNAs with the highest grade in *Y*^∗^ are more likely to be associated with the disease. The flow chart of MFMDA is shown in Figure 1.

## Results

### Evaluation of Prediction Performance

There are many performance indicators for evaluating prediction models. In this field, ROC curve and AUC value, PR curve, and AUPR value are usually used to evaluate the performance of the algorithm (Chen and Huang, 2017; Chen et al., 2020).

The ROC curve, also called receiver operating characteristic curve or susceptibility curve, is a comprehensive indicator reflecting sensitivity and specificity. The ROC curve graphically reveals the correlation between sensitivity and specificity. By setting different thresholds, a series of corresponding sensitivities and specificities are calculated, and then plotted with the true positive rate on the ordinate and false positive rate on the abscissa curve. The simple assumption is that for binary classification problems (only two types, positive and negative samples), the calculation methods of TPR and FPR are shown in Eq. 15.

(15) $$\text{TPR}=\frac{TP}{TP+FN},\mathrm{FPR}=\frac{FP}{TN+FP}$$

TP refers to the number of positive samples that are correctly predicted, that is, the number of positive samples that are predicted as positive samples; FP refers to the number of positive samples that are incorrectly predicted, that is, the number of negative samples that are predicted to be positive samples; the number of negative samples correctly predicted, that is, the number of negative samples predicted as negative samples; FN refers to the number of negative samples that are incorrectly predicted, that is, the number of positive samples predicted as negative samples. The area under the line of the ROC curve is AUC. The more convex the ROC curve, the closer to the upper left corner. The larger the AUC value, the better the prediction performance. The AUC value is generally between 0.5 and 1. The AUC value of 0.5 is the effect of random prediction. The AUC value of 1 has the best performance and the perfect classifier, that is, it can correct all positive and negative classes.

The PR curve calculates a series of accuracy and recall by setting different thresholds, and then draws the curve as the precision ordinate and recall as the abscissa. The precision and recall are calculated into the formulas 16:

(16) $$\text{precision}=\frac{TP}{TP+FP},\mathrm{FPR}=\frac{TP}{TP+FN}.$$

The PR curve reflects the correlation between accuracy and recall. The area under the PR curve is AUPR. The larger the AUPR value, the better the performance.

### Comparison With Other Methods

We further compared the prediction performance of the MFMDA model with four benchmark prediction models (i.e., LRMCMDA, IMCMDA, NCPMDA, and RLSMDA). LRMCMDA and IMCMDA belong to the matrix completion algorithm, and have achieved good predictive performance in this field. NCPMDA is a network projection algorithm, which is one of the representatives of algorithms based on network prediction. RLSMDA is a semi-supervised learning method based on the Regularized Least Squares (RLS) framework, which represents a good opportunity to learn learning algorithms. Since the data used in this study are all from the public data set HMDD2.0, all the parameters of the comparison algorithm will also use the parameters given by the original author.

### Performance on Predicting miRNA–Disease Association

**FIGURE 2:**
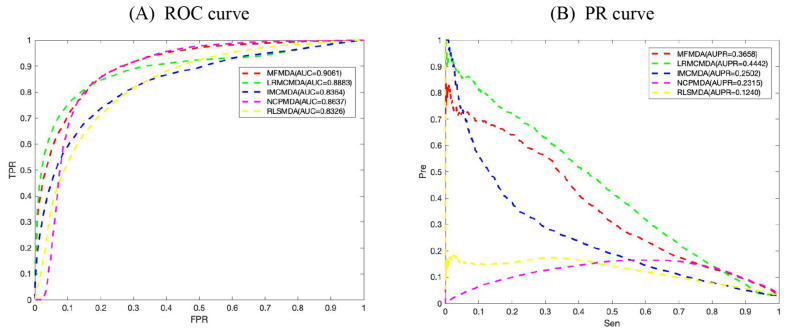
Comparison of MFMDA with four best performers for miRNA–disease associations. **(A)** ROC curves for fivefold cross validation. **(B)** Precision–recall (PR) curve for fivefold cross validation.

We applied MFMDA, LRMCMDA, IMCMDA, NCPMDA, and RLSMDA to HMDD V2.0 miRNA–disease association data, which contains 5430 unique associations between 495 miRNAs and 383 diseases, and draws their ROC curves of the global fivefold CV in Figure 2A. As can be seen, the AUCs of MFMDA, LRMCMDA, IMCMDA, NCPMDA, and RLSMDA are 0.9061, 0.8883, 0.8364, 0.8637, and 0.8326, respectively, indicating that MFMDA performed best in predicting miRNA–disease associations.

However, considering the limited number of known and experimentally verified miRNA–disease associations, it is too arbitrary to use AUC to evaluate the performance of prediction methods. Therefore, we also include the exact recall (PR) curve and the AUPR in Figure 2B to supplement performance evaluation. As shown in Figure 2B, the AUPR of MMFDA, LRMCMDA, IMCMDA, NCPMDA, and RLSMDA are 0.3658, 0.4442, 0.2502, 0.2315, and 0.1240, which again shows that MFMDA performs better than most algorithms in predicting miRNA–disease associations and can be a supplement to the existing computational prediction model.

### Predicting Novel Disease-Related miRNAs

**FIGURE 3:**
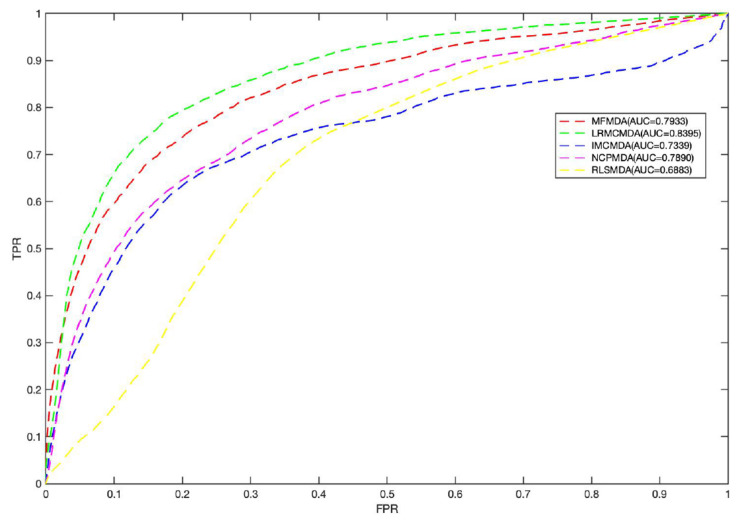
Comparison between MFMDA and benchmark algorithms based on local LOOCV.

For a new disease, if it can find its related miRNAs, it will provide a great help for people to understand the pathogenesis of the disease. Therefore, we performed *CV*_*d*_ experiment to test the performance of MFMDA in predicting miRNAs associated to a novel disease *d*. In *CV*_*d*_: CV on disease *d*_*i*_, we remove all the known miRNA–disease association of the disease *d*_*i*_ (column vectors in matrix *Y* ∈ *R*^*m*×*n*^) and build prediction model (for inferring the deleted associations) using the remaining data. As shown in Figure 3, the AUC value obtained by MFMDA is second only to LRMCMDA, which also indicates that MFMDA is also relatively good at predicting miRNAs related to new diseases. Of course, although LRMCMDA is more effective at predicting new disease-related miRNAs, LRMCMDA uses network projection to construct negative samples. This method of constructing negative samples will be affected by the size of the data set, which will affect its prediction performance. Presumably, MFMDA is a semi-supervised algorithm, it does not need to construct negative samples and the prediction performance is relatively stable.

Finally, we explored the effect of the disease similarity and miRNA similarity on prediction performance. Specifically, we performed global fivefold CV with parameters λ_*m*_or λ_*d*_ from 0.2 to 1 and a step size of 0.2 (Table 1). We can see that the two similarities really help predict performance. However, as the parameters continue to increase, the performance of the prediction is constantly decreasing.

**TABLE 1:** Prediction AUCs of MFMDA at different choices of parameters.

MFMDA	λ_*m*_ = λ_*d*_ = 0.2	λ_*m*_ = λ_*d*_ = 0.4	λ_*m*_ = λ_*d*_ = 0.6	λ_*m*_ = λ_*d*_ = 0.8	λ_*m*_ = λ_*d*_ = 1
AUC	0.9061	0.9058	0.9013	0.8924	0.8912

### Case Study

Next, three disease case studies were conducted to further validate the predictive power of the new miRNA disease pairs discovered by MFMDA. We first use the verified HMDD V2.0 pair as a training sample. For each predicted disease, the corresponding unverified miRNA is ranked according to the predicted score. Then, according to the other three well-known databases dbDEMC2.0 (Yang et al., 2017), miR2Disease (Jiang et al., 2009), and HMDD V3.0 (Huang et al., 2019b), the top 10 candidate miRNAs in the prediction list were examined.

Endometrial cancer is a group of epithelial malignant tumors that occur in the endometrium, and it occurs in perimenopausal and postmenopausal women. Endometrial cancer is one of the most common tumors of the female reproductive system. There are nearly 200,000 new cases each year, and it is the third most common gynecological malignant tumor that causes death. Earlier studies have shown that the differential expression of miRNA in endometrial adenocarcinoma can play a key auxiliary role in understanding the diagnosis and treatment of endometrial adenocarcinoma (Jurcevic et al., 2014). Therefore, in this study, we used MFMDA to identify potential miRNAs associated with endometrial adenocarcinoma. Nine of the top 10 miRNAs found were confirmed by at least one external database (see Table 2).

**TABLE 2:** The top 10 potential miRNA candidates detected by MFMDA for endometrial neoplasms.

Cancer	No. of confirmed miRNAs	Top 10 ranked predictions					
		Rank	miRNAs	Evidences	Rank	miRNAs	Evidences
Endometrial neoplasms	9	1	hsa-mir-146a	HMDD V3.0	6	hsa-mir-34a	HMDD V3.0
		2	hsa-mir-221	Unconfirmed	7	hsa-mir-29a	HMDD V3.0
		3	hsa-mir-20a	HMDD V3.0	8	hsa-mir-145	HMDD V3.0
		4	hsa-mir-17	HMDD V3.0	9	hsa-mir-15a	HMDD V3.0
		5	hsa-mir-16	HMDD V3.0	10	hsa-mir-29b	HMDD V3.0

In the second case study, we still choose the tumor that belongs to women with high incidence, namely, breast tumor. Breast tumors are malignant tumors that occur in the epithelial tissue of the breast glands. Currently, the treatment is mainly based on clinical and pathological features. Targeted therapy and personalized therapy are the ultimate goals. Related studies have shown that the occurrence of breast tumors is also related to abnormalities of related miRNAs. For example, an abnormal increase in miR-22 may promote the occurrence and metastasis of breast cancer and lead to a higher degree of tumor malignancy. Therefore, predicting miRNAs related to breast tumors through related algorithms will also provide corresponding help for human breast cancer treatment. As shown in Table 3, we found that the top 10 miRNAs predicted by MFMDA related to breast cancer have all been confirmed by relevant databases.

**TABLE 3:** The top 10 potential miRNA candidates detected by MFMDA for breast neoplasms.

Cancer	No. of confirmed miRNAs	Top 10 ranked predictions					
		Rank	miRNAs	Evidences	Rank	miRNAs	Evidences
Breast neoplasms	10	1	hsa-mir-150	dbDEMC 2.0	6	hsa-mir-130a	dbDEMC 2.0
		2	hsa-mir-142	dbDEMC 2.0	7	hsa-mir-99a	dbDEMC 2.0
		3	hsa-mir-15b	dbDEMC 2.0	8	hsa-mir-196b	dbDEMC 2.0
		4	hsa-mir-106a	dbDEMC 2.0	9	hsa-mir-378a	dbDEMC 2.0
		5	hsa-mir-192	dbDEMC 2.0	10	hsa-mir-212	dbDEMC 2.0

Finally, we conduct prediction studies on miRNAs associated with lung tumors. Lung cancer is one of the fastest growing morbidity and mortality rates, and the most threatening to the health and life of the population. In the past 50 years, many countries have reported that the incidence and mortality of lung cancer have increased significantly. The incidence and mortality of lung cancer in men accounted for the first place in all malignant tumors, the incidence in women accounted for the second place, and the mortality rate took the second place. Despite the important therapeutic value of chemotherapy, surgery is still the only way to treat lung cancer. There is an urgent need to find potential biomarkers that respond strongly to clinical observations. The researchers found that the expression level of miR-99a is related to the clinicopathological factors of lung cancer and lymph node metastasis. Identifying more miRNAs related to lung cancer helps to accurately assess clinical outcomes. Therefore, we conducted a lung cancer case study based on MFMDA. In the prediction list, nine of the top 10 predicted miRNAs confirmed their association with lung tumors (see Table 4).

**TABLE 4:** The top 10 potential miRNA candidates detected by MFMDA for lung neoplasms.

Cancer	No. of confirmed miRNAs	Top 10 ranked predictions					
		Rank	miRNAs	Evidences	Rank	miRNAs	Evidences
Lung neoplasms	9	1	hsa-mir-16	miR2Disease	6	hsa-mir-141	miR2Disease
		2	hsa-mir-122	dbDEMC 2.0	7	hsa-mir-195	miR2Disease
		3	hsa-mir-15a	dbDEMC 2.0	8	hsa-mir-429	miR2Disease
		4	hsa-mir-15b	Unconfirmed	9	hsa-mir-23b	dbDEMC 2.0
		5	hsa-mir-106b	dbDEMC 2.0	10	hsa-mir-20b	dbDEMC 2.0

**FIGURE 4:**
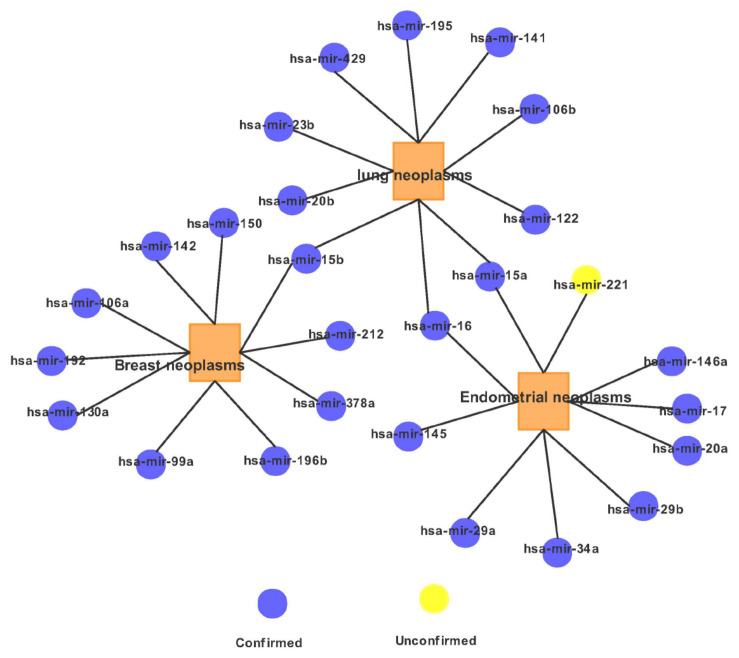
Network of the top 10 predicted associations for the three diseases via MFMDA.

For a clear view, we illustrate in Figure 4 the association network of the top 10 predicted miRNA candidates for the three diseases. It is worth noting that some top candidates were found to be related to several diseases. For example: hsa-mir-15a has not only been shown to be related to the occurrence of endometrial neoplasms, but also has a certain relationship with lung neoplasms.

## Discussion

A large number of studies have shown that miRNA plays an increasingly important role in many physiological processes. Researchers are trying to identify disease-related miRNAs as valuable biomarkers that can be used for clinical measurement, diagnosis, prognosis, and treatment. Therefore, accurately inferring potential miRNAs related to diseases can help us study the pathogenesis of diseases and find more effective treatments. In this study, we proposed a mathematical model based on MF (MFMDA) to identify potential miRNA–disease associations. First, MFMDA not only uses known miRNA and disease-related data, but also integrates the similarities between miRNA and disease. Second, the model is a semi-supervised model, which does not rely on negative samples. Finally, in the process of solving the model, we use the alternating gradient descent algorithm to find the optimal solution to ensure a stable decomposition matrix. Experimental results show that, compared with other methods, MFDMA can effectively improve performance and is a powerful tool for discovering the association of potential diseases with miRNA. However, this method still has some limitations; we need to further optimize. For example, the similarity measure between diseases and miRNAs used by MFMDA is too single and may not be the best choice. How to integrate multiple omics information more effectively to improve prediction performance is also worthy of further research.

## Data Availability Statement

The original contributions presented in the study are included in the article/supplementary material. Further inquiries can be directed to the corresponding authors.

## Author Contributions

SH and RC designed the study. PS collected and wrote the manuscript. SY and YC reviewed the manuscript. All authors contributed to the article and approved the submitted version.

## Conflict of Interest

The authors declare that the research was conducted in the absence of any commercial or financial relationships that could be construed as a potential conflict of interest.

## Footnotes

1 http://www.cuilab.cn/files/images/cuilab/misim.zip
